# Preparing for Life After Birth: Introducing the Concepts of Intrauterine and Extrauterine Sensory Entrainment in Mammalian Young

**DOI:** 10.3390/ani9100826

**Published:** 2019-10-18

**Authors:** David J. Mellor

**Affiliations:** Animal Welfare Science and Bioethics Centre, School of Veterinary Science, Massey University, Palmerston North 4474, New Zealand; d.j.mellor@massey.ac.nz; Tel.: +64-21-390-855

**Keywords:** fetal sensory systems, in utero sensory environment, sensory maturity at birth, trans-natal sensory continuity, sensory development after birth, learning, memory, neuroplasticity

## Abstract

**Simple Summary:**

A key event in the life of a mammalian fetus is its birth, especially in view of the exceptional change in its environment that occurs at birth. An area of great interest is the extent to which factors within the uterus prepare the fetus for birth and postnatal life. These and other factors are evaluated here for mammalian young that exhibit mature, moderately immature, and exceptionally immature neurological development at birth. A striking finding is the basic uniformity of various preparatory processes despite the diversity of birth-related circumstances among different terrestrial mammals. Numerous scientific disciplines have contributed to understanding in this area. Accordingly, the major purpose of this review is to construct from these diverse sources an integrated account of important facets of our current understanding. The primary focus is on how a progressive improvement in the functional effectiveness of the sensory systems for touch, temperature, taste, smell, balance/movement, hearing, and sight equips young mammals to cope successfully with the considerable challenge of being expelled from the uterus into the outside world. It is shown that the sensory environment in the uterus interacts with the genetically preprogrammed development of the apparatus for each sensory modality. It is also shown that the way this occurs can embed stimulus-response capabilities that can be accessed and beneficially utilised after birth. This phenomenon is called “trans-natal sensory continuity”. This embedding of sensory capabilities has previously been described as “learning” and the subsequent accessing of these embedded capabilities as “memory”. However, it is noted that such “learning” and “memory” processes are sometimes mistakenly thought to require the fetus to be conscious, a view which does not accord with established understanding of learning processes, nor with compelling contrary evidence from direct studies of fetal brain states. The solution proposed is to describe trans-natal sensory continuity in terms of the neurophysiological mechanisms involved and not as learning and memory outcomes. Introduced here, therefore, is the concept of “intrauterine sensory entrainment”. Its specific mechanistic basis is the well-established way nervous systems respond to sensory stimuli by reorganising their neural structures, functions, and connections. Known as neuroplasticity, this is recognised as integral to both prenatal and postnatal brain development and as the basis of postnatal learning and memory. Thus, intrauterine sensory entrainment is the mechanism by which trans-natal sensory continuity is achieved. It begins with the prenatal embedding of responsiveness to particular sensory inputs which are generated by the stimulation of specific receptors once the neurological apparatus for that sensory modality has begun to develop. Thereafter, this process continues until birth, and this makes all such entrained sensory capabilities available to operate after birth. Nevertheless, all sensory systems that exhibit some level of functionality at birth continue to mature postnatally, and in some species various sensory systems do not become functional until after birth. Accordingly, also introduced here is the concept of “extrauterine sensory entrainment”, which is the postnatal continuation of “intrauterine sensory entrainment”. Such extrauterine entrainment therefore contributes to the continuing maturation of the sensory systems that are operational at birth, the later development and maturation of those systems that are absent at birth, and the combined impact of these factors on the behaviour of newborn and young mammals.

**Abstract:**

Presented is an updated understanding of the development of sensory systems in the offspring of a wide range of terrestrial mammals, the prenatal exposure of those systems to salient stimuli, and the mechanisms by which that exposure can embed particular sensory capabilities that prepare newborns to respond appropriately to similar stimuli they may encounter after birth. Taken together, these are the constituents of the phenomenon of “trans-natal sensory continuity” where the embedded sensory capabilities are considered to have been “learnt” and, when accessed subsequently, they are said to have been “remembered”. An alternative explanation of trans-natal sensory continuity is provided here in order to focus on the mechanisms of “embedding” and “accessing” instead of the potentially more subjectively conceived outcomes of “learning” and “memory”. Thus, the mechanistic concept of “intrauterine sensory entrainment” has been introduced, its foundation being the well-established neuroplastic capability of nervous systems to respond to sensory inputs by reorganising their neural structures, functions, and connections. Five conditions need to be met before “trans-natal sensory continuity” can occur. They are (1) sufficient neurological maturity to support minimal functional activity in specific sensory receptor systems in utero; (2) the presence of sensory stimuli that activate their aligned receptors before birth; (3) the neurological capability for entrained functions within specific sensory modalities to be retained beyond birth; (4) specific sensory stimuli that are effective both before and after birth; and (5) a capability to detect those stimuli when or if they are presented after birth in ways that differ (e.g., in air) from their presentation via fluid media before birth. Numerous beneficial outcomes of this process have been reported for mammalian newborns, but the range of benefits depends on how many of the full set of sensory modalities are functional at the time of birth. Thus, the breadth of sensory capabilities may be extensive, somewhat restricted, or minimal in offspring that are, respectively, neurologically mature, moderately immature, or exceptionally immature at birth. It is noted that birth marks a transition from *intrauterine* sensory entrainment to *extrauterine* sensory entrainment in all mammalian young. Depending on their neurological maturity, extrauterine entrainment contributes to the continuing maturation of the different sensory systems that are operational at birth, the later development and maturation of the systems that are absent at birth, and the combined impact of those factors on the behaviour of newborn and young mammals. Intrauterine sensory entrainment helps to prepare mammalian young for life immediately after birth, and extrauterine sensory entrainment continues this process until all sensory modalities develop full functionality. It is apparent that, overall, extrauterine sensory entrainment and its aligned neuroplastic responses underlie numerous postnatal learning and memory events which contribute to the maturation of all sensory capabilities that eventually enable mammalian young to live autonomously.

## 1. Prologue

The major purposes of this review are to update, collate, integrate, and present a synthesis of information regarding the development of sensory systems in the offspring of a wide range of terrestrial mammals, the prenatal exposure of those systems to salient stimuli, and the mechanisms by which that exposure can embed particular sensory capabilities that prepare newborns to respond appropriately to similar stimuli they may encounter after birth. Another objective is to introduce the mechanistic concept of “intrauterine sensory entrainment”, briefly referred to in 2007 as “neurophysiological entrainment” by Mellor and Diesch [1], because this terminology is potentially less open to misinterpretation than are the notions of fetal “learning” and “memory”. Also introduced is the mechanistic concept of “extrauterine sensory entrainment”, which is a continuation of the entrainment occurring prenatally, but which has particular relevance to the impact of the markedly different sensory environment of the young during the continued maturation and/or development of their sensory systems after birth. 

## 2. Introduction

“The first day of life” is a term often mistakenly used in the literature to mean “the first day after birth” (e.g., [2,3,4,5,6,7,8]). Its use is misleading because viable newborns have obviously been alive from conception to the end of gestation. Thus, birth represents the transition from intrauterine *life* to extrauterine *life*, and much is now known about this transition. Some specific areas of greatly improved understanding are as follows: the fetal mechanisms that initiate labour and their additional role in the maturation of fetal tissues essential for postnatal survival; prenatal physiological and pathophysiological factors that jeopardise newborn survival; cardio-vascular and cardio-respiratory changes at birth; the synchronisation of birth and the onset of lactation; the transition from fetal unconsciousness to postnatal consciousness; survival-critical metabolic responses to postnatal thermal challenges; and mother–young interactions required for successful rearing of offspring in diverse species-specific natal circumstances (for references see [1,9,10,11,12,13,14,15,16,17,18,19,20,21,22,23,24,25]).

Other studies have focused on the development and roles of specific sensory systems, their responses to particular stimuli at each developmental stage, and the ways their responses at each stage are influenced by, and are related to, those of preceding and subsequent stages [26]. Significant attention has been given to trans-natal phenomena, that is, key features of the transition from prenatal stages to the immediate postnatal stage of development (e.g., [27,28,29,30,31,32,33,34,35,36,37]).

Obviously, any sensory system that possesses some level of functional capability immediately after birth, however mature or immature that functionality might then be, would have developed that capability before birth. Equally, particular stimuli present in utero have the potential to influence the development of the related sensory apparatus in ways that embed particular response outputs, so that if encountered postnatally these stimuli will elicit the preprogrammed responses and thereby influence neonatal behaviours. In the cases where this is known to occur, it has been characterised as trans-natal gustatory (taste), olfactory (smell), or auditory (sound) continuity, where the prenatal embedding of specific response capabilities has been characterised as “learning” and the postnatal accessing of that functionality has been considered to represent “memory” (e.g., [5,30,38,39,40,41,42,43,44,45]) (see Section 5). 

The overall approach adopted here parallels that of a related, but distinctly different, exploration of other survival-critical perinatal phenomena [25]. As with that discussion, the information utilised here was published over several decades but only rarely in the animal science or veterinary literature. Rather, it was distributed widely in behavioural neuroscience, biomedical science, comparative physiology, developmental biology, developmental psychobiology, ethology, neonatology, and perinatology journals. This review is therefore directed towards scientists with broad interests in comparative developmental physiology and perinatology, as also to other animal-based scientists, including veterinarians, who wish to gain an updated, integrated, and more detailed understanding to support their management of a wide range of newborn and young mammals.

This review has the following structure. First, the sensory capabilities of newborn mammals that exhibit different levels of neurological maturity are revealed by briefly describing their behaviour immediately after birth (Section 3). Second, the in utero sensory environment of the embryo/fetus and its potential impacts on prenatal sensory development are considered (Section 4). Third, different forms of postnatal learning and memory are briefly described (Section 5) to provide background for the next section. Fourth, focusing on the mechanism underlying “trans-natal sensory continuity” is recommended as being preferable to the potentially misleading concepts of fetal “learning” and “memory” (Section 6). Fifth, the concept of “intrauterine sensory entrainment”, achieved via neuroplastic responses of the fetal nervous system to specific sensory inputs, is introduced as the mechanism underlying “trans-natal sensory continuity” (Section 7). Sixth, the role and significance of the transition at birth from “intrauterine sensory entrainment” to “extrauterine sensory entrainment” are discussed (Section 8). Finally, the review ends with concluding comments (Section 9).

## 3. Neurological Maturity in Newborn Mammals and What Their Behaviour Reveals about Which Sensory Systems Are Functional at Birth

In general, for a sensory system to become sufficiently functional to generate a conscious experience of its particular modality, operational connectivity is required between modality-specific receptors and their peripheral, spinal, and brainstem nerve pathways, or their dedicated cranial nerves, which then direct sensory impulse traffic to specific subcortical and cortical regions of the brain for the processing that gives rise to each sensation (e.g., [34,35,36,37,38,39,40,41,42,43,44,45,46,47]). Note, however, that cortically supported conscious experience of, and cognitively directed responses to, particular sensations in mammalian young cannot occur before the establishment of neural connectivity between the cerebral cortex and the subcortical regions of the brain; note also that the timing of when this connectivity occurs in relation to birth depends on the neurological maturity of the young at birth (for references see [25,35]) (also see Section 3.1).

Different sensory modalities become functional in most mammalian young by following a common developmental sequence. This begins with the somatosensory system (touch, temperature) and continues with the chemosensory systems (taste, smell), nociceptive system (pain), vestibular and proprioceptive system (balance, motion, position), and the auditory system (hearing), and ends with the visual system (for references see [25,26,47,48,49,50,51]). Evidence for this has been provided by studies of the developmental anatomy and histology of specific receptors, nerve pathways and their associated organs, as also neurochemical, electrophysiological, and behavioural responses associated with each sensory modality.

### 3.1. Newborn Mammals Exhibit Three General Levels of Neurological Maturity

Three groups of young mammals may be identified on the basis of their level of neurological maturity at birth: *neurologically mature* newborns, for example, the offspring of primates, guinea pigs, and ungulates such as cattle, deer, goats, sheep, horses, and pigs; *neurologically moderately immature* newborns, for example, the offspring of bears, cats, dogs, ferrets, hamsters, mice, rats, and rabbits; and the *neurologically exceptionally immature* newborns of marsupials such as kangaroos, wallabies, and opossums (for references see [16,25]). 

Major criteria of such maturity include a progression in the electrical activity in the embryonic/fetal cerebral cortex (see [20,25,52]). Initially there is no activity, then it appears as short epochs which expand to become continuous, and, once continuous, it develops into distinct mature patterns. This last mature stage coincides with the establishment of the neural cortical–subcortical connections referred to above, connections which appear before birth in fetuses that are born neurologically mature and at about 10–17 days or 2–6 months afterwards in young that are neurologically moderately or exceptionally immature at birth [25,35].

In accord with the above observations on electrocortical activity, levels of neurological maturity are also reflected in the extent to which the common pattern of sensory development has progressed by the time of birth in each group; in other words, which sensory modalities are and are not operational at birth, and how long it takes postnatally for all of the modalities to become functionally effective [35]. This is an important stage in neurological maturation because only when all sensory modalities are functioning can the young flexibly deploy the full range of survival-enhancing behaviours of which they are then capable during interactions with their dams, littermates (if any), and environment (for references see [25]). As already indicated, the postnatal onset of such behavioural flexibility in these three groups occurs after several minutes-to-hours (mature), days-to-weeks (moderately immature), and months (exceptionally immature) [25].

To secure their survival during the intervening period, maternal care and protection must compensate for the behavioural inadequacies that result from the limited sensory capabilities of their offspring [25]. In the *neurologically mature group*, apart from pigs (see below), this usually involves focused maternal attentiveness facilitated by the rapid establishment of an exclusive mother–young bond with her 1–3 offspring; in the *moderately immature group*, birth and rearing of 4–10 or more young usually occurs in a burrow, den, nest, or other enclosed area which facilitates maternal care and protection of the litter as a group; and in the *exceptionally immature group*, 1–12 joeys (marsupial young) rapidly enter the maternal pouch, attach to a teat or teats, and thereafter are carried and assiduously nurtured and protected by the mother [18,25,53].

### 3.2. Sensory Capabilities and Behaviour of Neurologically Mature Newborns

Immediately after birth, ruminant newborns rapidly engage in complex interactive bonding behaviours with their dam, including standing, walking, and searching for the maternal udder [18,54]. These behaviours rely on touch, proprioception, balance, thermal sensitivity, smell, taste, hearing, and sight [18,28,55,56,57,58,59]. For this to occur, these sensory modalities must have developed before birth, despite the character and intensity of stimuli met in utero often being markedly different from those encountered after birth [19,49]. For example, the fetus is buoyant and cushioned from shock in amniotic fluid, its movements are restricted by the uterus, and its body temperature is kept just above that of the mother. In contrast, the newborn has its first experience of non-cushioned gravity, air, hard surfaces, unlimited space, and, usually, cold challenge [19,21,49].

Piglets exhibit similar sensory maturity at birth, which in natural circumstances usually takes place in a nest constructed by the mother [18,58,60]. Without assistance from the mother, newborn piglets follow her body surface to the ventrum, locate the udder and a teat, and begin to suck [18]. The required sensory capabilities include proprioception and vestibular function for standing and walking, hearing for detecting maternal grunts, olfaction to detect udder-sourced odours, thermal sensitivity for seeking warmth, touch for detecting the direction of abdominal hair growth and contact of the snout with protruding teats, and sight for returning to the udder and recognising littermates [56,60,61,62]. Notwithstanding their sensory maturity, piglets are born with an immature thermoregulatory capability, but this is counterbalanced by the nest which provides shelter and facilitates huddling between litter mates and the mother while their thermoregulation improves during the first seven postnatal days [63,64].

Most sensory capabilities of newborn human infants are also advanced, but some are less mature than in ungulates. Tactile sensitivity in the lips and oral cavity supports sucking from a nipple; also, such receptors in the hands enable grasping and, distributed over the thorax, abdomen, and limbs, they underlie a capability to experience soothing touch [48,65,66,67]. Musculoskeletal control, proprioception, and vestibular function are immature at birth as movement is usually restricted to reflexive rooting, sucking, startle, grasp, and arm, leg, and neck movements [68,69]. Olfactory detection of areolar, breast, and milk odourants guides nipple location and supports the onset of sucking [70,71,72], and taste discrimination underlies preferences for breast milk as opposed to formula and sweet as opposed to sour or bitter tastes [41,67,73]. The nociceptive system is operational and generates experiences of pain in response to noxious stimulation [20,46,66], and hearing is sufficiently mature to be responsive to a wide range of sounds including parental and other voices, music sequences, and startling noises [30,74,75]. Finally, vision is somewhat immature at birth [34], indicated by uncoordinated eye movements, a limited ability to track objects, and a capability to focus only on objects at close range, specifically at 18 to 25 cm [66,67]. However, this short focal capability may be functionally appropriate because it would correspond to the distance between the mother’s face and the baby in her arms [67].

### 3.3. Sensory Capabilities and Behaviour of Neurologically Moderately Immature Newborns

The behaviour of newborns in this group includes crawling towards the mother, locating her mammary glands, orally grasping a nipple and sucking, thermoregulatory huddling with littermates and the dam, and eliciting maternal nurturing and protective behaviours [76,77,78]. These and other behaviours provide clear evidence that particular sensory modalities are sufficiently functional at birth to enable the litter to attain and maintain close proximity with the dam; they include proprioception, touch, taste, smell, thermal sensitivity, and nociception [36,52,60,78,79,80,81,82]. Also, a degree of taste discrimination is apparent immediately after birth [83]. However, hearing and sight are absent and, depending on the species, do not become functional until cortical–subcortical connectivity is established at about 10–17 days after birth [25,34,76,77,78]. 

### 3.4. Sensory Capabilities and Behaviour of Neurologically Exceptionally Immature Newborns

Exceptional neurological immaturity is common to all marsupial joeys at birth [50,84]. For example, a Tammar wallaby (*Macropus eugenii*) joey is born after a 28-day gestation with a cerebral cortex which consists of only two cell layers and resembles that of a 26-day sheep or 40-day human embryo [85]. Nevertheless, unaided by the dam, it immediately climbs from the urogenital sinus into her pouch where it locates and then orally attaches itself to a teat [86,87,88]. Clearly, the joey’s pouch orientating, climbing, teat locating, and oral grasping behaviours provide evidence of responsiveness to inputs from rudimentary sensory modalities that are operational at birth but which do not include nociception, hearing, and sight. However, they do include gravitational, olfactory, gustatory, and thermal sensory inputs [89,90,91] and inputs from tactile sensory receptors in the muzzle and around the mouth [92,93]. This reflects the very early stages of the cephalocaudal developmental pattern of the somatosensory system, observed in most developing mammals, whereby responsiveness to touch first appears at the mouth, lips and face or snout, then extends caudally to the trunk, followed thereafter by the proximal and then the distal regions of the limbs [26,46,48,49,66]. Note that marsupial pouch young are dependent on maternal milk for all sustenance during the period of pouch occupancy, which lasts for about 190 days in the Tammar Wallaby [87]. Neurologically they mature slowly. It takes 160–180 days for their tactile, thermal, proprioceptive, nociceptive, olfactory, and, lastly, their auditory and visual systems to mature sufficiently for them to temporarily exit the pouch for the first time at about 190 days of age (for references see [25]).

### 3.5. Comment

It is obvious that any sensory system that possesses some level of functional capability immediately after birth, however mature or immature that functionality might then be, begins its development before birth. Also, it is known that exposure to some quite specific stimuli in utero can affect the operational development of the sensory system in question by embedding equally specific response capabilities that persist through birth, thereby enabling them to be accessed by the newborn [24,33]. Accordingly, the sensory environment of the embryo/fetus merits detailed consideration.

## 4. The Intrauterine Sensory Environment of the Embryo/Fetus

Contrary to earlier views that the embryo/fetus occupies a sensory void in utero (for references see [48]), it is now well established that there exists a wide range of stimuli that have demonstrable impacts on sensory systems as they develop (e.g., [5,30,38,39,40,41,42,43,44,46,48,49,66]).

### 4.1. Cutaneous Senses

#### 4.1.1. Tactile Stimulation

The embryo/fetus remains fully immersed in amniotic fluid held within the membranous amniotic sac until birth. Initially, the fluid volume is high in relation to body size so that the embryo/fetus floats freely within the sac and is well cushioned against potentially harmful buffeting (e.g., sheep [94,95,96,97]). Amniotic fluid volume increases thereafter until about mid-pregnancy, keeping pace with fetal size. However, as fetal growth continues the ratio reverses, cutaneous contact with the amniotic membrane increases, and fetal movements become more restricted (e.g., sheep [19,21,97]; human [98]). This, together with limb movements, squirming, and turning, as well as physical contact with littermates (if any), provides opportunities for significant tactile stimulation. Indeed, body stretching and other limb, trunk, and neck movements total 4000–6000 per day in the fetal lamb during the last 14 days of pregnancy (for references see [19]).

#### 4.1.2. Thermal Stimulation

Fetal cutaneous thermoreceptors will likely be stimulated over a narrow range because the environment in utero is thermally stable and fetal temperature remains 0.5–1.5 °C above maternal temperature (e.g., sheep [99,100,101]). Heat produced by the fetus is lost down that temperature gradient and is dissipated by the mother’s overriding thermoregulatory capability. Notwithstanding this exposure to a narrow range of temperatures in utero, cutaneous thermoreceptors demonstrably develop the capability to elicit metabolic responses to cold challenge, both shortly before birth when fetal lambs are experimentally cooled in utero [102,103,104,105] and immediately after birth when newborns are exposed to ambient cold (e.g., lambs [106]; lambs, piglets, and human infants [12]; lambs, kids, calves, foals, and piglets [107]).

#### 4.1.3. Nociceptive Stimulation

Fetal cutaneous nociceptors would likely receive minimal stimulation during pregnancies that progress without traumatic, therapeutic, or pathological injury to the skin. Yet, these nociceptors clearly develop functionally in utero because invasive skin manipulations elicit physiological stress and other responses when conducted both before birth (e.g., fetal lambs and fetal human infants [20,46,108,109]) and soon after birth (e.g., lambs [110]; piglets [111]; lambs, calves, piglets [112]; human infants [46,66,108]). Of course, variable levels of fetal nociceptor stimulation are likely to occur as a result of labour-induced compression and/or injuries sustained during normal and, especially, difficult births (e.g., [17,19,21,113]).

### 4.2. Gustatory Sense

Fluid dynamics of relevance to gustatory stimuli in mammalian fetuses are complex. Sheep fetuses, for example, swallow a fluid mixture which contains substances that can stimulate oral taste receptors [114,115,116]. These substances, usually sourced from the dam’s diet, are carried to the placenta in her blood. After crossing the placenta, they are transported throughout the fetus in its blood, and may therefore enter any secretions produced by the fetus [37]. The fluid swallowed by the fetus is a mixture of fetal urine voided into the amniotic sac, fetal lung fluid actively secreted into the mouth, fetal saliva, and, possibly, fluid entering the amniotic sac across the fetal membranes and, depending on the species, across the skin (e.g., sheep, goat, human, and rat [37,114,115,116,117,118,119,120,121,122,123,124,125,126,127,128,129,130,131]). After being swallowed, the fluid is then absorbed from the gut lumen into the fetal blood. In addition, these substances may stimulate the oral taste receptors after being delivered via the blood into the tissues around them [37,132].

Generally, fetal taste buds develop morphologically in advance of their functionality, such that at birth they exhibit an operational capability that increases on a maturational trajectory that continues after birth [41,47], for example, in lambs [133,134], human infants [45,48,66,135], puppies [136], and kittens [137]. In light of their relative neurological maturity at birth (Section 3), however, it may be speculated that the taste sensing capability in newborn lambs and infants might be greater than that in newborn puppies and kittens [48,133,134,135,136,137].

### 4.3. Olfactory Sense

The dynamic fluid interactions and tissue blood flows responsible for delivering taste-related substances to receptors in the fetal oral cavity (Section 4.1.2) probably also deliver fluid-borne and blood-borne aromatic substances to olfactory receptors in various distinct areas within the two nasal cavities of the fetus, including the main olfactory region and the vomeronasal organ [37]. However, the olfactory potential in the human fetus is likely to be impeded until about 28 weeks of pregnancy, which is when plugs in the nasal cavities dissolve [138]. This then enables fluid to flow through the nasal cavities during fetal swallowing and breathing [115,116] and soluble odourants to access the nasal receptors [45]. Some of these odourants may originate from the dam’s diet or, if volatile, from inhaled air [37]. Moreover, other odourants may be synthesised by the dam in genetically preprogrammed combinations that furnish her amniotic fluid with a unique aromatic signature [66]. Prenatal exposure to these odourants, together with others that reflect the specific dietary choices of the dam, likely explain why newborn infants prefer their own amniotic fluid to that from another mother [139,140,141]. As with taste receptors, fetal olfactory receptors develop morphologically in advance of their functionality [48], and at birth they exhibit significant operational capabilities that continue to mature after birth [47], for example, in human infants [37,41,45] and lambs [37,54,142]. 

### 4.4. Vestibular Sense

Located in the inner ear, the vestibular system generates proprioceptive information which is essential for well-coordinated movements, balance, and posture [143]. More specifically, it coordinates eye movements in relation to the position of the head and detects rotational motion (angular acceleration) and head orientation relative to vertical gravitational forces (linear acceleration). Its inputs are closely linked to the function of the cerebellum and to reflex processes in the spinal cord and brain stem which together are responsible for well-integrated movements of eyes, head, neck, trunk, and limbs. 

It is obvious that the vestibular system achieves a high level of functionality before birth in those neurologically mature newborns that engage in well-coordinated ambulatory, udder-searching, and other maternally directed behaviours immediately after birth, but proportionately less functionality in human infants (Section 3.2). Thus, compared to the vestibular functionality achieved in most newborns in this mature group, the levels of functionality in the newborns categorised as neurologically moderately immature (Section 3.3) and exceptionally immature (Section 3.4) are, respectively, lower and much lower. It is apparent that throughout intrauterine development the fetal vestibular apparatus, whatever functional level it achieves at birth, is exposed to potentially formative stimuli every time the mother and/or fetus move. Although there is little direct experimental support for this reasoned conclusion, some relevant contextual information is available (e.g., [33,48,49,144,145,146,147]).

### 4.5. Auditory Sense

It is apparent from hearing-related neonatal behaviour that the auditory apparatus develops a high degree of functionality in mammalian young which are neurologically mature at birth, including human infants (for references see [25,27,29,33,34,48,49,148,149]). This contrasts markedly with neurologically moderately immature young in which the postnatal onset of functional hearing is delayed for several days-to-weeks [76,77,78] and with exceptionally immature newborns in which hearing onset is delayed for several months [150]. Note for these groups that this is associated with corresponding delays in the establishment of cortical–subcortical connections within the brain [24,25].

These observations suggest that the sound environment in utero is likely to have the most impact on the developing auditory apparatus of those embryos/fetuses that will be neurologically mature at birth. Natural and/or artificial sounds having different acoustic characteristics, detected in utero using hydrophones placed near the fetal head, have been shown to elicit auditory evoked potentials, heightened metabolic activity in hearing-related cerebral areas, and/or heart rate and musculoskeletal responses in guinea pigs, goats, sheep, and, receiving the most attention, human beings (for references see [27,29,30,40,43,48,49,66,148,149,151,152,153,154,155]).

In utero background noise is generally of low intensity, so that, for example, it does not mask the maternal voice which forms a prominent part of the fetal sound environment in both sheep [152,156,157,158,159] and human beings [29,30,49,155]. Speaking, reciting, singing, playing music, or generating additional sounds externally elicits auditory-related responses in human fetuses, but often less effectively due to potential sound attenuation at the maternal air–skin interface [29,39,49,66]. In contrast, the internal generation of maternal vocal sound waves, which directly resonate within her body, including the uterine contents, apparently has an enhanced capability to stimulate the fetal auditory apparatus (for references see [30,40,66,160,161]). In addition, the auditory apparatus in utero is continuously exposed to the specific, potentially formative characteristics of the mother’s voice every time she vocalises.

### 4.6. Visual Sense 

It is apparent from sight-related neonatal behaviour that the visual apparatus develops a high degree of functionality in those young which are neurologically mature at birth, except for human infants (for references see [18,25,28,48,55,56,57]). Nevertheless, the range of visual capabilities in newborn infants includes blinking, responsiveness to light, controlled ocular motility, object fixation, detection and tracking, focusing on close objects, and object recognition whether stationary or moving (for references see [34,66,162]). These capabilities develop independently of specific visual stimuli in utero, except for some variations in low-level light intensity [34,49]. In marked contrast, the onset of sight occurs postnatally in the neurologically moderately immature young after several days-to-weeks [48,76,77,78] and after several months in the exceptionally immature newborns [87,163]. As with hearing, the late onset of functional sight in these groups is associated with corresponding delays in the establishment of cortical–subcortical connections within the brain [25,164,165].

### 4.7. Interoceptors, Exteroceptors, and Genetic Preprogramming of Sensory Functions

Two broad categories of sensory receptors have been recognised [166,167]. *Interoceptors* detect stimuli from within the body, for example, thermoreceptors (brain temperature), osmoreceptors (solute levels in body fluids), baroreceptors (blood pressure), and chemoreceptors (O_2_, CO_2_, pH). *Exteroceptor*s detect stimuli originating from outside the body, for example, cutaneous mechanoreceptors (touch), thermoreceptors (temperature), and nociceptors (pain), as well as chemoreceptors (taste, smell), proprioceptors (gravity), vibroreceptors (sound), and photoreceptors (sight). The prenatal development of the specific detection-related functionality of each receptor within its sensory system is genetically preprogrammed [34]. Thus, as sensory systems develop on their preprogrammed trajectories, interoceptors begin to respond to stimuli generated within the embryo/fetus, and exteroceptors start to respond to different stimuli that originate from within the dam or, via the dam, from her environment (Section 4.1, Section 4.2, Section 4.3, Section 4.4, Section 4.5 and Section 4.6). Note that each sensory system apparently has a genetically preprogrammed capability to become responsive to the range of aligned stimuli to which it may be exposed in utero, for example, to a range of taste compounds (e.g., [45,73]), a range of odour compounds (e.g., [134,168]), and a range of sound stimuli (e.g., [29,30,34,40]). Note also that such preprogramming is apparently sufficient in itself to achieve high levels of postnatal functionality in sensory systems even when prenatal stimulation of the salient receptors appears to remain quite limited [34], a point illustrated by the above references to effective operation of the thermal, nociceptive, and visual systems in neurologically mature newborns (Section 4.1.2, Section 4.1.3 and Section 4.6). 

## 5. A Brief Introduction to Aspects of Learning and Memory

The branch of biology that focuses on the neurological foundations of thinking, feeling, learning, memory, and other such brain functions that influence the nature of the subjective experiences human beings and other animals may have, and the ways those experiences may be expressed behaviourally, has variously been called psychobiology, psycho-neurology, behavioural neuroscience, and other similar names [26,169]. In this subject area, “learning” is considered to have occurred when a change in behaviour takes place as a result of direct experience [5]. Each learned experience depends on the sensory modality or modalities that provide significant inputs for the required brain processing to occur (see Section 3 and [5,49]). Such inputs therefore contribute to the content of what is learnt. 

Importantly, learning and memory are mutually dependent functions, because something that cannot be actively or passively accessed via memory cannot be said to have been learnt. Moreover, retention of memories ranges from being short-lived to long-lasting, with evidence for their continued existence being the persistence of novel, distinctive, or changed behaviours after learning has occurred [5,49]. Such behaviours include observable features of animals’ physical appearance, demeanour, activity, and/or vocalisation, as well as measurable elements of their internal physiological responses.

Different types of learning have been identified and categorised as “associative” and “non-associative” [5,49,170]. Varieties of associative learning are said to include “imprinting” or “exposure learning”, “classical conditioning”, and “operant conditioning”, whereas “habituation” or “desensitisation” is a form of simple non-associative learning (Table 1). All of these learning varieties proved to be informative when applied beyond the experimental context that revealed them (e.g., [170,171,172,173,174,175,176]).

Thus, psychobiologists explore different facets of the mutually dependent functions of learning and memory by reference to changes in behaviour elicited by brain processing of different sensory inputs. As already noted, behaviour in this context is considered to include that which is observable externally as well as indicative internal physiological changes. Investigated for the most part in conscious animals, these external and internal categories of behaviour can both be interpreted as suggestive of a wide variety of conscious subjective experiences which are meaningfully linked to learning and memory. Note, however, that the neurophysiological mechanisms, including the brain processing that is primarily responsible for eliciting overt behaviours and any associated conscious experiences, all operate below the level of consciousness (Table 1). This distinction is relevant to the present consideration given to the prior development of those sensory capabilities that underlie “learning” and “memory” functions which are considered to be operational at birth (Section 6 and Section 7).

## 6. Trans-natal Sensory Continuity and Potential for Misinterpreting the Concepts of Fetal “Learning” and “Memory”

### 6.1. Trans-natal Sensory Continuity

Trans-natal *sensory* continuity is a generic term introduced here and potentially includes all sensory modalities. However, three of them have received detailed attention. They are trans-natal *gustatory* continuity [41,184], *olfactory* continuity [35,37,38,41,184], and *auditory* continuity [30,39,40,43,44]. Such continuity results from prenatal stimuli which affect the operational development of their fetal sensory systems by embedding particular response capabilities that persist beyond birth, thereby potentially influencing the behaviour of the newborn. This occurs when the aligned exteroceptors for each modality are exposed, via the dam, to specific combinations of taste, smell, or sound stimuli which are products of her unique genetics and/or are specific features of her environment.

Three human examples, among many in the literature, will suffice to illustrate this. The first relates to the mother’s environment, in particular the transmission of flavour and/or odourant compounds from her diet to the fetus (Section 4.2 and Section 4.3). These compounds have been shown to heighten the newborn infant’s preferences for the same tastes and smells found in the mother’s milk, thereby enhancing the onset of breast feeding [35,37,39,41,184]. Second, specific combinations of maternal areola pheromones (presumably products of genetically embedded processes) and maternal dietary odorants on her breast skin combine to further facilitate breast feeding by guiding nipple searching, oral grasping, and the onset and continuation of sucking by the infant [70,71,72]. Finally, positive responses of newborn infants to the mother’s voice, transmitted internally within her body throughout the prenatal development of the fetal auditory apparatus (Section 4.5), help to secure the wider benefits of mother–infant bonding very soon after birth [5,30,39,40,45,185,186,187].

### 6.2. Potential Misinterpretation of the Concepts of Fetal “Learning” and “Memory”

As already mentioned, learning and memory appear to be mutually dependent functions because that which cannot be accessed through memory, actively or passively, cannot be said to have been learnt (Section 5). In the cases where trans-natal gustatory, olfactory, and/or auditory continuity have been demonstrated, the prenatal embedding of specific response capabilities has been characterised as “learning” and the postnatal accessing of that functionality has been considered to represent “memory” (e.g., [5,30,33,37,38,39,40,41,42,43,44]). In other words, fetuses are understood to be capable of “learning” the sensory processing of particular stimuli in ways that enable that processing to be accessed or “remembered” and thus utilised beneficially by the newborn. Furthermore, fetal responses to the postnatal learning paradigms of exposure learning, classical conditioning, and habituation (Section 5, Table 1) provide additional evidence that fetuses do indeed have a capability for such “memory” processing (for references see [33,37,39,155]).

Developmental psychobiologists understand that conscious processing of sensory inputs is not required for fetal “learning” and “memory” to occur (e.g., [5,26,37,38,39,49]). Moreover, they understand that a significant proportion of postnatal learning and memory also occurs below the level of consciousness (e.g., [188,189], Section 2). However, as most people are only able to recall learning and memory activities of their own that required focused attention, in the absence of this specialist understanding, they often think that consciousness must be involved for the required brain processing to occur both before and after birth. Compounding this misapprehension is the persistence of a view that human fetuses are conscious during late pregnancy (e.g., [190,191,192,193,194]). Factors that contribute to this view include erroneous interpretation of early recordings of electroencephalographic activity in sheep fetuses (e.g., [195,196,197]) and of ultrasound observation of human fetal behaviour states (e.g., [109,198,199,200,201,202,203]), both of which have been taken to suggest that periods of arousal or wakefulness occur during at least the last one-third of gestation (see [20]). However, there is now compelling evidence that at least six in utero neuroinhibitory factors actively keep both sheep and human fetuses in continuous states of unarousable, sleep-like unconsciousness, so that they cannot consciously experience any sensations prior to birth [1,20,21,46,108,113]. These factors have been demonstrated to have inhibitory actions of the fetal brain. They include *adenosine*, a potent inhibitor of fetal electrocortical activity; *pregnanolone* and *allopregnanolone,* synthesised by the fetal brain, which have anaesthetic, sedative, and analgesic actions; *prostaglandin D_2_*, a potent sleep-inducing hormone; at least one *placenta peptide* having neuroinhibitory effects; neuro-suppressive effects of *warmth;* and combined effects of *buoyancy* and *cushioning* from soft tissues that limit tactile stimulation [1,16,20,21].

### 6.3. The Underlying Mechanism of Trans-natal Sensory Continuity Needs to be Emphasised

These observations are not intended to cast doubt on the occurrence of what has to date been described as trans-natal gustatory, olfactory, or auditory continuity involving fetal “learning” and “memory” processing; rather, their purpose is to highlight a need to revise the associated terminology to avoid implying that fetuses are conscious of any related subjective experiences. Particular words, and their variants, which it is suggested should be avoided when referring to specific sensory inputs in embryos/fetuses include learning, memorising, familiarising; memory, remembering, recognising, experiencing, feeling, sensing; tasting, smelling, hearing, and seeing. Note that many of them may be found in the literature on this subject (e.g., [30,33,34,35,37,39,40,41,42,43,44,45,74,155,191]). The preferred alternative approach, therefore, is to reformulate the explanations in terms of the physiological mechanisms involved in trans-natal gustatory, olfactory, and auditory continuity (generalised here as “trans-natal sensory continuity”) by introducing the concept of “intrauterine sensory entrainment”, previously referred to as “neurophysiological entrainment” [1].

## 7. Trans-natal Sensory Continuity, Intrauterine Sensory Entrainment, and Neuroplasticity

### 7.1. Trans-natal Sensory Continuity

Trans-natal sensory continuity is the phenomenon whereby exposure to particular sensory inputs before birth establishes a capability for the young to respond to the same sensory inputs immediately after birth. This may occur through activation of both interoceptors and exteroceptors. Five conditions must be met for trans-natal sensory continuity to occur (adapted from a specific example of trans-natal olfactory continuity [37]). They are (1) sufficient neurological maturity to support minimal functional activity in specific sensory receptor systems in utero; (2) the presence of sensory stimuli that activate their aligned receptors before birth; (3) the neurological capability for entrained functions within specific sensory modalities to be retained beyond birth; (4) specific sensory stimuli that are effective both before and after birth; and (5) a capability to detect those stimuli when or if they are presented after birth in ways that differ (e.g., in air) from their presentation via fluid media before birth. The literature in this area reveals numerous examples where all five conditions are met.

### 7.2. Intrauterine Sensory Entrainment and Neuroplasticity

Intrauterine sensory entrainment is the process by which trans-natal sensory continuity is achieved. It begins with the prenatal embedding of responsiveness to particular sensory inputs which are generated by the stimulation of specific receptors once the neurological apparatus for that sensory modality has begun to develop. Thereafter, this process continues until birth, and this makes all such entrained sensory capabilities available to operate after birth. Thus, for example, postnatal responsiveness specifically to the maternal voice is envisaged to be due to intrauterine entrainment of impulse barrages associated with often-repeated sounds occurring to an extent that, after birth, newborn human infants can distinguish those sounds from others not previously registered by its auditory apparatus at all, as often, as loudly, or in the same patterns.

The mechanistic basis of intrauterine sensory entrainment is neuroplasticity. This is the fundamental property of nervous systems which enables intrinsic or extrinsic stimuli to reorganise neural structures, functions, and/or connections, for example, during prenatal and postnatal brain development and as a basis for postnatal learning and memory [34,204,205,206]. Thus, in utero stimulation of sensory receptors apparently initiates activity-dependent development and maintenance of neuronal pathways having specific functional characteristics supported by dynamically aligned features of, for example, synaptic strength, efficacy, and transmission; axonal structure; and dendritic branching [34,204,205,206]. Such intrauterine neuroplastic activity does not require the involvement of consciousness to occur and, as illustrated in the previous paragraph, may be described using terms that do not reference or infer subjective experiences.

## 8. The Transition from “Intrauterine” to “Extrauterine” Sensory Entrainment

Most of the above examples of trans-natal sensory continuity, and, thus, intrauterine sensory entrainment, have been drawn from those young that become neurologically mature by the time of birth [25]. Note however that such trans-natal continuity and its aligned intrauterine entrainment are also known to occur in the young of cats, dogs, rabbits, and rats (e.g., [32,37,38,52,72,79,80,136,137,207]), which are representative of young that are neurologically moderately immature at birth [25]. No such observations could be found for marsupial joeys which are born neurologically exceptionally immature [25]. This may be because their small size and pouch occupancy make them technically too difficult to study. Note, however, that the rudimentary sensory capabilities newborn joeys are known to possess (Section 3.4) would in any case likely be expressive of an early stage in the genetically preprogrammed development of their sensory systems [34].

Birth marks a transition from *intrauterine* sensory entrainment to *extrauterine* sensory entrainment during the progressive neurological development that occurs in all mammalian young. The postnatal roles and continuing maturation of the different sensory systems that are operational at birth, the later development and maturation of the systems that are functionally absent at birth, and their combined influence on the behaviour of newborn and young mammals in these three groups have been described briefly here (Section 3) and in detail elsewhere [25]. The markedly different environments encountered after birth would obviously alter the balance and possibly the character of exteroceptor inputs to the sensory systems functioning at birth. This in turn would induce specific neuroplastic responses at the onset of extrauterine sensory entrainment which, at birth and subsequently, would contribute to the distinctive postnatal behavioural changes that occur according to the different time scales observable in the three groups [25].

Whereas intrauterine sensory entrainment helps to prepare mammalian young for life after birth, within the wider context of extrauterine entrainment, gustatory and olfactory entrainment, for example, may have specific roles in preparing the young for life after weaning. This is because their complete initial dependency on the dam’s milk for sustenance ensures that delivery of the flavour and odourant compounds specific to her dietary choices will continue after birth. This would help to guide the non-milk dietary choices of the young as they approach and then pass beyond weaning. Note from a safety viewpoint that entrained preferences for attractive dietary tastes and smells (e.g., [73,208,209]), and genetically preprogrammed aversion to others [41,48,208,210], would likely act together to minimise consumption of potentially toxic compounds.

Finally, it is apparent that extrauterine sensory entrainment and its aligned neuroplastic responses are essential constituents of many learning and memory events which are integral to the development of those sensory capabilities that eventually enable mammalian young to live autonomously. Additional specific examples of this include the various postnatal learning/memory scenarios outlined in Table 1.

## 9. Concluding Comments

It is apparent that the genetically preprogrammed functional development of the apparatus for each sensory modality, interacting dynamically with the sensory environment at each stage, determines which sensory systems are operational in mammalian young both before and after birth. It also determines the extent to which earlier exposure to particular stimuli may enhance the responsiveness of the apparatus to those specific sensory stimuli during later stages. This is highlighted by clarification of the mechanistic basis of “trans-natal sensory continuity” in terms of “intrauterine sensory entrainment” which involves stimulus-induced neuroplastic changes to brain structures, functions, and/or connections. Such neuroplasticity continues postnatally as the mechanistic foundation of “extrauterine sensory entrainment”. Thus, such entrainment contributes to completing the maturation of all sensory systems which are present at birth and, depending on the species, also to the development and maturation of those systems that only begin to function after birth. It is recommended that such mechanistic explanations be used in preference to the terms fetal “learning” and “memory”. Note, however, that neuroplasticity is also the foundation of well-demonstrated postnatal learning and memory functions and that this becomes especially apparent after neural connectivity within the brain enables cortically supported conscious experience of, and cognitively-directed behavioural responses to, particular sensations.

## Figures and Tables

**Table 1:** Brief descriptions of imprinting or exposure learning, of classical conditioning and operant conditioning as examples of associative learning, and of habituation or desensitisation as a form of non-associative learning.

Imprinting or Exposure Learning	Classical Conditioning	Operant Conditioning	Habituation or Desensitisation
This is any kind of phase-sensitive learning that occurs at a particular age or life stage, is rapidly acquired, and is apparently independent of the consequences of behaviour. Expressed generically, an animal or person learns the characteristics of some stimulus which is then said to be imprinted on the subject. Once imprinted, exposure to the stimulus elicits particular behaviours that are likely associated with consciously perceived subjective experiences. A well-known form is “filial imprinting”. This occurs where a young animal narrows its social preferences to an object (typically a parent) as a result of exposure to that object. This is obvious in the hatchlings of birds such as waders, water fowl, and gamebirds, which imprint on their parents, then follow them around [170] and when separated make “distress calls” [177,178]. Similar behaviours are observable in newborn mammals such as lambs, kids, calves, fawns, and foals, which need to remain near their mobile mothers [14,18,107,179]. Thus, imprinting is a learning mechanism that likely contributes to rapid mother–young bonding as occurs in neurologically mature hatchlings (not discussed here) and newborn mammals (Section 3).	Also known as Pavlovian conditioning, this form of associative learning typically involves repeatedly pairing an unconditioned stimulus, which evokes a specific response, with another previous neutral stimulus, which does not evoke that response [171,180]. When the stimuli are presented separately after conditioning has taken place, the response occurs both to the unconditioned stimulus and to the other unrelated (now conditioned) stimulus. In Pavlov’s historic study, initially a dog presented with meat salivated but did not do so when a bell rang. After conditioning, which involved repeatedly inducing the dog to salivate by presenting meat and then ringing a bell, both stimuli, presented separately, caused the dog to salivate. Classical conditioning involves automatic or reflexive responses, and thus the underlying mechanisms operate below the level of consciousness [180]. Although the operation and linking of these mechanisms is involuntary, the subjective elements of the resulting behavioural responses are experienced consciously. For example, Pavlovian conditioning has been implicated as contributing to the establishment and maintenance of a range of social behaviours, including sexual behaviour, mother–young bonding, maternal suckling and lactation, and social grooming, where their attendant subjective elements are undoubtedly experienced consciously [171].	This method of learning occurs through animals consciously associating “rewarding” (i.e., “reinforcing”) or “punishing” outcomes with particular behaviours. Thus, pleasant or unpleasant consequences inform choices about which behaviours are preferred or avoided, the former usually being retained and the latter rejected or modified [172,173]. Accordingly, investigations of such learning focus on behaviours that can be changed because of their specific experiential outcomes. Application of the principle that “reinforcement” and “punishment” can influence conscious behavioural choices extends well beyond the laboratory setting as such learning often also plays a powerful role in everyday learning [176]. Detailed accounts of various operant conditioning methodologies, their wide applications, and observed neurobiological correlates are available elsewhere [172,173,176].	This is an example of non-associative learning in which there is a progressive diminution of a behavioural response with repetition of a stimulus, such that, following an initial response to the stimulus, the frequency and/or strength of the subsequent responses diminish with repeated stimulation [170,174]. As noted above, the response may be detected by overt behavioural changes and/or internal physiological reactions. For example, pregnant ewes brought from outdoor field conditions into unfamiliar housing and placed individually in adjacent pens, when handled initially, invariably exhibited aggressive, escape, and/or excitable behaviour [181]. However, after 6–8 weeks of daily gentle handling for 5–10 minutes this behaviour was replaced by calm, relaxed, and even positive responses when staff approached. These overt signs of habituation were paralleled by a progressive decline in the cortisol stress and heartrate responses to handling from high initial values to no detectable change [181]. Subsequently, in another laboratory, gentling of wethers was also shown to reduce their heart rate, flight distance, and aversion responses to handling [182]. Regarding the first case, undertaken as a pre-study taming and training protocol for pregnant ewes [181,183], initially the hands-on close proximity of the staff was obviously aversiveness, but as habituation progressed the animals often came forward to greet staff when they entered the pen and then nuzzled them during the physical contact. This suggests that habituation learning predominated at first [174]. However, elements of operant conditioning might also have contributed in those animals that eventually elicited further gentle stroking by nuzzling the staff, stroking which arguably then represented a form of positive reinforcement [173].
